# A New Species of the Genus *Pseudocalotes* (Squamata: Agamidae) from Southwest Yunnan, China

**DOI:** 10.3390/ani14060826

**Published:** 2024-03-07

**Authors:** Yuhao Xu, Yanan Gong, Mian Hou, Shiyang Weng, Shuo Liu, Jundong Deng, Junkang Hu, Lifang Peng

**Affiliations:** 1 State Key Laboratory of Plateau Ecology and Agriculture, Qinghai University, Xining 810016, China; yuhao_xu@sinoophis.com (Y.X.); junkang@sinoophis.com (J.H.); 2 Anhui Province Key Laboratory of the Conservation and Exploitation of Biological Resource, College of Life Sciences, Anhui Normal University, Wuhu 241000, China; yan_an_gong@sinoophis.com; 3 College of Continuing (Online) Education, Sichuan Normal University, Chengdu 610068, China; turtlechina@126.com; 4 Tibet Plateau Institute of Biology, Lhasa 850008, China; wisely@sinoophis.com; 5 Kunming Natural History Museum of Zoology, Kunming Institute of Zoology, Chinese Academy of Sciences, Kunming 650223, China; liushuo@mail.kiz.ac.cn

**Keywords:** *Pseudocalotes jingpo* sp. nov., molecular systematics, morphological characters, taxonomy, lizard

## Abstract

**Simple Summary:**

In this study, a new species of the genus *Pseudocalotes* is described from Yingjiang County, Dehong Dai, and Jingpo Autonomous Prefecture, Yunnan Province, China, based on four female specimens. Phylogenetic analyses based on *NADH dehydrogenase subunit* 2 (ND2) and *NADH dehydrogenase subunit* 4 (ND4) indicated that the new taxon is different from its congeners. Morphologically, the new species can be diagnosed from the other species by a combination of 18 characters. The recognition of this new species brings the number of described *Pseudocalotes* species to 22.

**Abstract:**

In this study, a new species of the genus *Pseudocalotes* is described from Yingjiang County, Dehong Dai and Jingpo Autonomous Prefecture, Yunnan Province, China, based on four female specimens. It can be distinguished from its congeners by the following combination of characters: (1) interoculabials 3 or 4; (2) canthals 5–7; (3) cicrcumorbitals 8–11; (4) 1 scale between rostral and nasal; (5) interparietal 1; (6) superciliaries 4–6; (7) supralabials 6–7, the 1st in contact with the nasal; (8) infralabials 6–8; (9) transverse gular fold and antehumeral fold present; (10) 2–3 enlarged scales between eye and ear; (11) nuchal crest single, consists of 3–5 erected spines; (12) dorsal crest row single, discontinuous and low, located between two keeled, parallel and enlarged scale rows; (13) enlarged postrictals absent; (14) scales around midbody 53–62, dorsal body scales heterogenous in size and shape; (15) midventrals smaller than dorsals; (16) subdigital scales on the 4th finger 20–26, and on the 4th toe 24–29; (17) dorsal background coloration light taupe with four irregular brown patches along the middle of dorsal; (18) inner lips wathet, tongue aurantiacus, throat bluish black. The population from Yingjiang County was nested within a highly supported lineage, formed a sister taxon with *P. kakhienensis* (SH 97/UFB 100) and according to the p-distance, the new species differed from its congeners by 14.5% to 35.2% for *NADH dehydrogenase subunit* 2 (ND2) and 15.5% to 25.0% for *NADH dehydrogenase subunit* 4 (ND4).

## 1. Introduction

The agamid lizards of the genus *Pseudocalotes* Fitzinger, 1843, which type species is *P. tympanistriga* (Gray, 1831), represents a mountainous, arboreal group that are widely distributed in South to South East Asia, from southern China, Laos, Myanmar, Vietnam, Thailand, Cambodia to the Malay Peninsula and major landmasses of the Sunda Shelf [1,2,3,4,5,6,7,8]. Currently, it contains at least 21 recognized species, of which 4 are found in China: *P. brevipes* (Werner, 1904), *P. kakhienensis* (Anderson, 1879), *P. kingdonwardi* (Smith, 1935), and *P. microlepis* (Boulenger, 1888) [8,9].

Yingjiang County lies in southwest Yunnan Province, geographically at the southeastern-corner of the Tibetan Plateau and at the southern-tip of the Hengduan Mountains, which is the most affected by the southwest tropical monsoon in China. Within the territory, numerous mountains, wide valleys, and rivers intersect with each other, forming several climatic zones and resulting in the regional divergences of species and high biodiversity. However, compared with other areas with high biodiversity, this region has not been surveyed in details for herpetological diversity. So, the species diversity in this region might still be underestimated.

**Figure 1:**
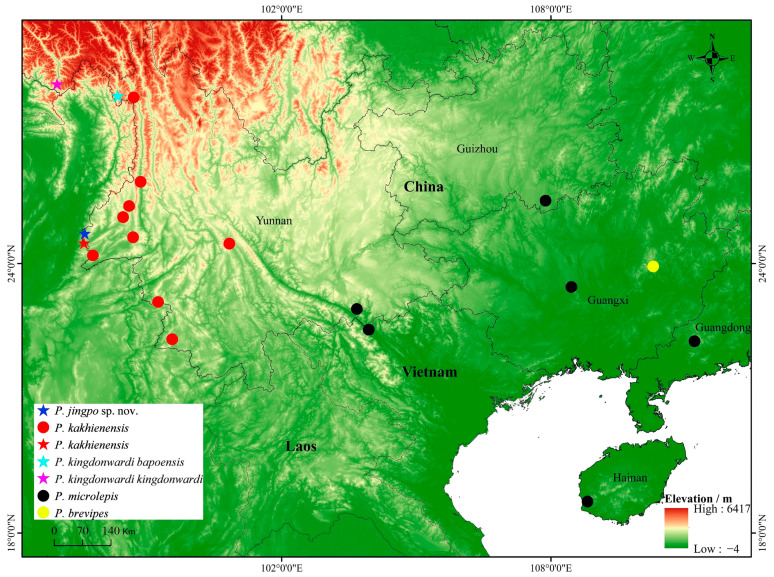
Known distribution of species of the genus *Pseudocalotes* in China: *Pseudocalotes jingpo* sp. nov. (dark blue pentacle), *P. kakhienensis* (red pentacle and red circle), *P. kingdonwardi kingdonwardi* (pink pentacle), *P. kingdonwardi bapoensis* (wathet pentacle), *P. microlepis* (black circle), and *P. brevipes* (yellow circle). Pentacles represent the type locality, and circles represent the known localities.

During a herpetological survey in Yingjiang County, Dehong Dai and Jingpo Autonomous Prefecture, Yunnan Province, China (Figure 1) in November 2023, we collected four lizard specimens. Although they could be assigned to *Pseudocalotes* by having distinctively small scales of central gular region; dorsal scales heterogenous in size and shape; smooth scales of the lateral head; absent post-orbital and post-occipital spines; elongated and tall nuchal crest scales; the middle suborbital scale row significantly enlarged; and feeble and low dorsal crest scales, but they cannot be identified as any known species morphologically [3,4,7]. Furthermore, molecular analyses supported by these specimens comprise a separate evolution lineage. Thus, we described the specimens from Yunnan Province, China as a new species.

## 2. Material and Methods

### 2.1. Sampling

Three adult and one subadult lizard specimens were collected from Yingjiang County, Yunnan Province, China. Sex was determined by dissection of the specimens, and determining if ovaries were present. The specimens were humanely euthanized using a lethal injection of 0.7% tricaine methanesulfonate (MS222) solution. Fresh liver tissue was extracted and immediately preserved in 95% ethanol. The specimens were fixed in 10% formaldehyde, then transferred to 75% ethanol for permanent preservation, and deposited at the Qinghai University Museum. Sampling procedures involving live lizards were conducted in accordance with the Wild Animals Protection Law of China.

### 2.2. Molecular Phylogenetic Analyses

Genomic DNA were extracted from preserved liver tissues using QIAamp DNA Mini Kit (QIAGEN, Changsheng Biotechnology Co., Ltd., Changchun, China). Fragments of the *NADH dehydrogenase subunit* 2 (ND2) were obtained by polymerase chain reaction (PCR) using the primers (5′-CCACCAAACAACTACACCTA-3′)/Jap_1559R (5′-GGATTAATGCCCTCTGGATT-3′) in ND2 [7] and “ND4” (5′-CACCTATGACTACCAAAAGCTCATGTAGAAGC-3′)/“LEU” (5′-CATTACTTTTACTTGGATTTGCACCA-3′) in ND4 [6]. PCR products were sequenced by Shanghai Map Biotech Co., Ltd., Shanghai, China. The raw sequences were assembled using SeqMan in the DNASTAR software package [10], and compared by MEGA X software [11]. The maximum likelihood (ML) was used IQ-TREE 1.6.12 [12] to construct the phylogenetic tree. Ultrafast Bootstrap Approximation (UFB) node support was assessed by using 5000 ultrafast bootstrap replicates and the UFB (%) ≥ 95 was considered significantly supported [13]. In addition, the single branch tests were conducted by SH-like approximate likelihood ratio test (SH-aLRT) by 1000 replicates and the nodal support (SH, %) ≥80 was also considered supported well [14]. Uncorrected pairwise distances (*p*-distance) among closely related congeners were calculated in MEGA X software [11]. The newly generated sequences were uploaded to GenBank.

To explore the phylogenetic relationships of the specimens from Yingjiang County, we used concatenated ND2 and ND4 sequences of 15 recognized species of the genus *Pseudocalotes*, and the homologous sequences of 3 recognized genera of Draconinae, including *Bronchocela*, *Dendragama*, and *Gonocephalus* in analysis. We also used the homologous sequences of *Pogona vitticeps* (Amphibolurinae) and *Calotes versicolor* (Draconinae) as outgroups (Table 1) [5,6,7,15,16,17,18,19,20,21]. All sequences, except the newly generated sequences, were obtained from the National Center for Biotechnology Information (NCBI).

### 2.3. Morphological Examination

Morphological characters were obtained from the newly collected specimens and many key references [1,2,3,4,5,6,7,22,23,24,25,26,27,28,29,30,31,32,33,34,35,36,37]. Measurements and scale counts were performed following Zhao et al., 1999, Harvey et al., 2014, Grismer et al., 2016 [4,5,34] and were developed using digital calipers to the nearest 0.1 mm.

Abbreviations for measurements are as follows: total length (TL); snout to vent length (SVL); tail length (TAL); head length from the tip of the snout to posterior axis of the jaw, where in front of the first scale of the nuchal crest (HL); head width at its widest point (HW); maximum head depth taken at the rear axis of the jaw (HD); forelimb length (FLL); and hindlimb length (HLL). Scalation features are as follows: interoculabials: the number of scales between supralabials and suboculars; postrostrals: the numbers of scales in contact with the rostral scale, excluding the supralabials; canthals: the number of enlarged scales between the postnasal and the anteriormost superciliary; circumorbitals: the number of enlarged scales extending from the anterior margin of the orbit and grading into larger scales that terminate at the posterior margin of the orbit, forming a semicircular arc; superciliaries: the number of flat, elongate, imbricating scales immediately above the orbit; supralabials: the number of scales extending from the rostral to just beyond the rictus; infralabials: the number of scales extending from the mental to the scales positioned beneath the last supralabial; postmentals: the number of scales in contact with the mental, excluding the infralabials; chin-shields: the number of enlarged scales between the mental that extend posteriorly along the margin of the mandible medial to the infralabials; gular scales: the number of scales between the postmentals and the prebrachial margin; nuchal crest: the number of elongated, lanceolate, vertebral scales on the nape of the neck; scales around midbody; Finger IV subdigital lamellae count: the number of subdigital lamellae scale from the base to the tip of Finger IV, excluding the claw; Toe IV subdigital lamellae count: the number of subdigital lamellae scales from the base to the tip of Toe IV, excluding the claw.

Other morphological characters considered important for taxonomic identification in the genus *Pseudocalotes* were recorded according to Harvey et al., 2014 and Grismer et al., 2016 [4,5]: the number of scales between the rostral and nasal; the number of supralabials in contact with the nasal; the number of enlarged scales between the eye and the ear; the number of enlarged posttemporals, supratympanics, and postrictals; the presence or absence of an interparietal scale and parietal eye; the presence or absence of transverse gular and antehumeral folds; the condition of preaxial lamellae of the third toe; the dewlap color pattern; and the presence or absence of a gular spot.

## 3. Results

### 3.1. Phylogenetic Relationship

**Figure 2:**
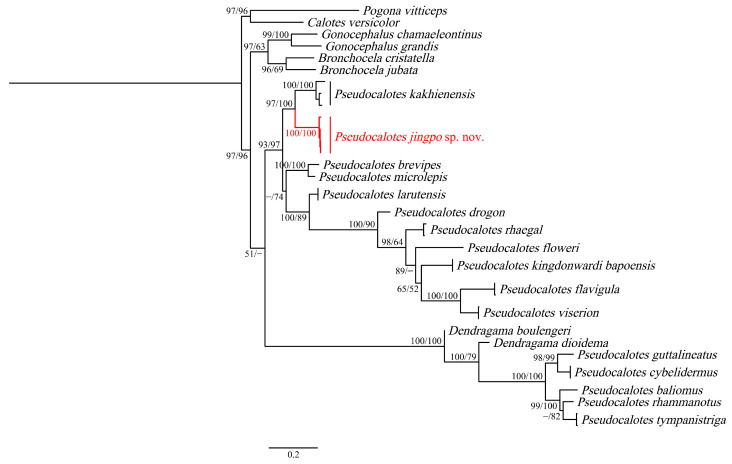
The phylogenetic relationship trees of *Pseudocalotes* based on both Maximum Likelihood (ML) of two mitochondrial fragments (ND2 and ND4). The values on the corresponding branches indicate SH/UFB, while the values under 50% are omitted. Tips for the new species in this study are shown in red.

All specimens (QHU2024001–QHU2024004) were successfully sequenced. The newly generated sequences were deposited in GenBank (Accession numbers: PP228248–PP228251 in ND2, and PP356075–PP356078 in ND4), corresponding voucher and collection number: QHU2024001, LFR2023033; QHU2024002, LFR2023040–QHU2024004, LFR2023042). The topology obtained by Maximum Likelihood analysis is shown in Figure 2, based on 1498 bp concatenated ND2 and ND4 gene sequences, indicates that the relationships of the genus is strongly supported: ((*Gonocephalus*, *Bronchocela*), *Pseudocalotes*). The genus *Pseudocalotes* is divided into two unrelated clades with low support: clade 1 undoubtedly includes the vast majority of species in the genus *Pseudocalotes*; but clade 2, which contains *P. Dioidema, P. guttalineatus*, *P. cybelidermus*, *P. baliomus*, *P*. *rhammanotus* and *P. tympanistriga*, is more closely related to the genus *Dendragama*. This result differs from the results obtained by Harvey et al., 2017 [6].

Meanwhile, our analyses clearly assigned the phylogenetic position of the newly collected specimens to the genus *Pseudocalotes*. Four specimens of the new species from Yingjiang County, Yunnan Province were nested within clade 1, and formed a sister taxon with *P. kakhienensis* (SH 97/UFB 100). According to the *p*-distance, the new species differed from its congeners by ranges from 14.5% (vs. *P. kakhienensis*) to 35.2% (vs. *P. tympanistriga*) in ND2 (Table 2) and from 15.5% (vs. *P. kakhienensis*) to 25.0% (vs. *P. tympanistriga*) in ND4 (Table 3). Moreover, morphological data also support the recognition of the specimens from Yingjiang County as distinct from all other described species of *Pseudocalotes*. Thus, we described the unnamed specimens as a new species.

### 3.2. Taxonomic Account

Squamata; Iguania; Agamidae; Draconinae; *Pseudocalotes*; *Pseudocalotes jingpo* sp. nov. XU, GONG, HOU, WENG, LIU, DENG, Hu, and PENG http://zoobank.org/urn:lsid:zoobank.org:act:FBF1A402-DF7F-44BC-878C-50C5548AD32B (accessed on 30 January 2024) Figure 3, Figure 4 and Figure 5.

**Figure 3:**
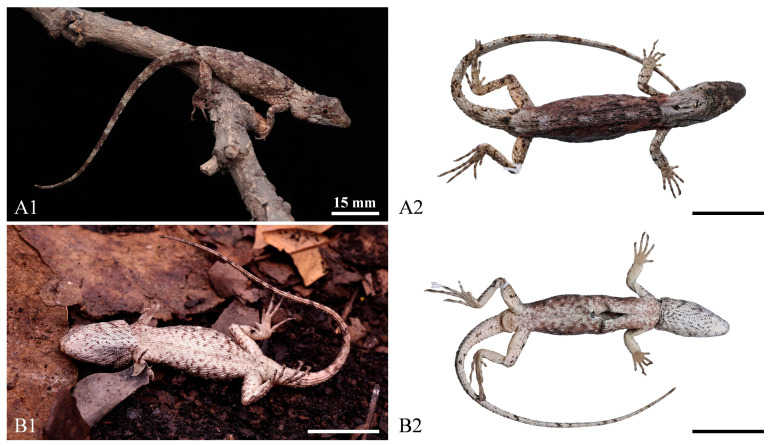
Dorsal view (**A1**,**A2**) and ventral view (**B1**,**B2**) of the holotype of *Pseudocalotes jingpo* sp. nov**.** (QHU2024002) in life (**A1**,**B1**) and in preservative (**A2**,**B2**). Scale bars: (**A1**–**B1**,**A2**–**B2**) = 15 mm.

**Figure 4:**
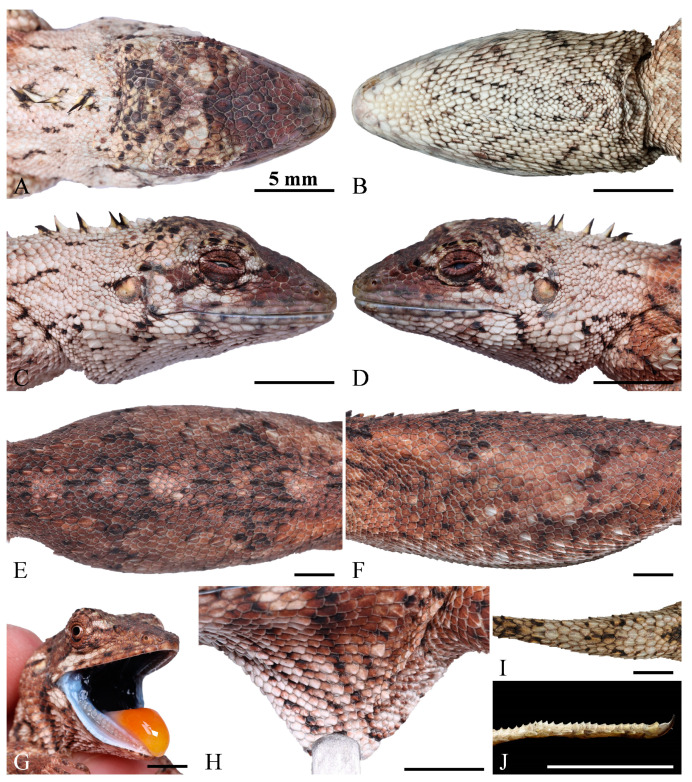
Dorsal (**A**), ventral (**B**), right (**C**), left (**D**) views of the head, dorsal (**E**), and lateral (**F**) view of body, close-up view of the oral cavity (**G**), dewlap (**H**), the base of dorsal tail (**I**) and the lateral view of preaxial scales on the third toe (**J**) of the holotype (QHU2024002) of *Pseudocalotes jingpo* sp. nov. Scale bars: (**A**–**J**) = 5 mm.

Holotype. QHU2024002 (field number LFR2023040, Figure 3 and Figure 4), adult female, collected by Jundong Deng and Lifang Peng on 27 November 2023 (24°40′13.44″ N, 97°36′37.44″ E, 970 m a. s. l.), Yingjiang County, Dehong Dai and Jingpo Autonomous Prefecture, Yunnan Province, China.

**Figure 5:**
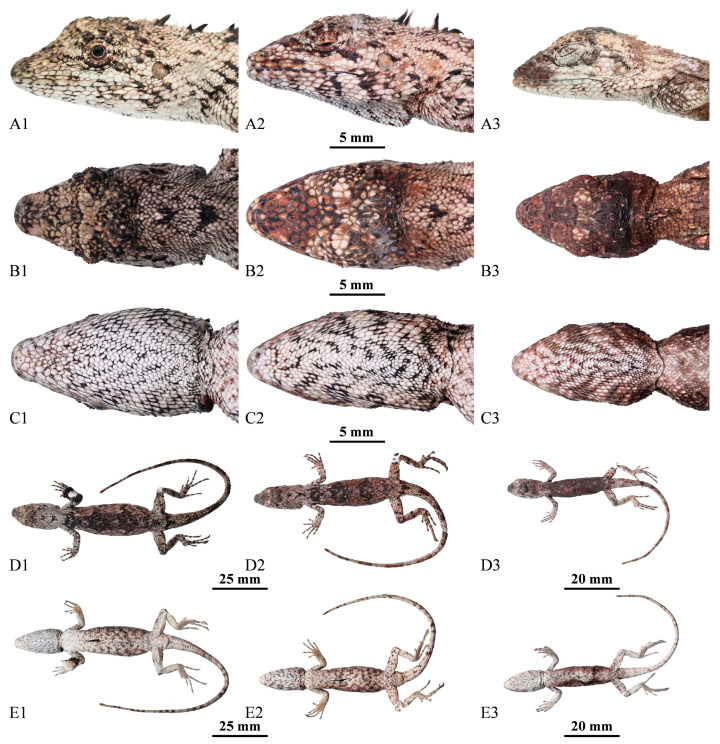
Lateral (**A1–A3**), dorsal (**B1–B3**), ventral (**C1–C3**) views of the head, general view of dorsal (**D1–D3**) and ventral (**E1–E3**) of the paratypes of *Pseudocalotes jingpo* sp. nov. (**A1**–**E1**): QHU2024001, adult female; (**A2**–**E2**): QHU2024003, adult female; (**A3**–**E3**): QHU2024004, subadult female. Scale bars are shown in the figure.

Paratypes. Two adult females: QHU2024001, QHU2024003 (field number LFR2023033, LFR2023041, Figure 5), and one subadult female: QHU2024004 (field number LFR2023042, Figure 5), all with the same collecting information as the holotype.

### 3.3. Diagnosis

*Pseudocalotes jingpo* sp. nov. can be distinguished from its congeners in the genus *Pseudocalotes* by the following combination of characters: (1) interoculabials 3 or 4; (2) canthals 5–7; (3) cicrcumorbitals 8–11; (4) 1 scale between the rostral and nasal; (5) interparietal 1; (6) superciliaries 4–6; (7) supralabials 6–7, the 1st in contact with the nasal; (8) infralabials 6–8; (9) transverse gular fold and antehumeral fold present; (10) 2–3 enlarged scales between the eye and ear; (11) nuchal crest single, consists of 3–5 erected spines; (12) dorsal crest row single, discontinuous and low, located between two keeled, parallel and enlarged scale rows; (13) enlarged postrictals absent; (14) scales around the midbody 53–62, dorsal body scales heterogenous in size and shape; (15) midventrals smaller than the dorsals; (16) subdigital scales on the 4th finger 20–26, and on the 4th toe 24–29; (17) dorsal background coloration light taupe with four irregular brown patches along the middle of dorsal; (18) inner lips wathet, tongue aurantiacus, throat bluish black.

### 3.4. Description of Holotype

Adult female, SVL 57.5 mm and TAL 85.3 mm, tail complete, TAL/SVL ratio 1.48; TAL/TL ratio 0.60; head relatively large, subtriangular in lateral and dorsal view, HL 15.1 mm, HW 8.9 mm, HD 8.9 mm, HW/HL ratio 0.59; FLL 12.7 mm, HLL 18.2 mm; finger IV > III > II > V > I and Toe IV > III > V > II > I in relative length; nostril lateral, round, piercing in the middle of the nasal, almost invisible in dorsal view.

Dorsal head scales keeled and slightly imbricated; rostral sloped anteriorly, visible dorsally, subhexagonal, about 2.5 times as wide as tall, in contact with postrostrals and the first supralabials; postrostrals 6, small, postrostral series separated the nasal and rostral; nasal subhexagonal, width almost equal to the height; canthus rostralis sharp, canthals 5/6, keeled; in prefrontal region, 6 enlarged, heavily keeled scales forming a Y-shaped series; scales of frontal region keeled, smaller than medial supraoculars; interparietal scale small, hexagonal and keeled, about twice as long as wide, surrounded by swollen and keeled scales; a small parietal eye in the center of interparietal; circumorbitals 11/12, keeled and relatively enlarged; supraciliaries 6/5, elongate except for last one; temporal scales of varying sizes and shapes, enlarged posttemporals 1/1; tympanum naked; supratympanic 1, swollen, keeled and slightly enlarged; enlarged scales between eye and ear 2/2, separated by one small scales; interoculabials displaying 4 complete rows between supralabials and suboculars; supralabials 7/7, smooth, the 1st supralabial in contact with the nasal; infralabials 7/8, smooth; postmentals 4, the outer 2 relatively enlarged, contacting infralabials and forming the first of chin-shields; chin-shields 5/4, separated from infralabials by one anteriorly and two posteriorly rows of small scales; gular scales small and smooth, slightly thickened, with the tip directed posteromedially, 53 at midline; gular pouch well developed, the transverse gular fold present.

The nuchal crest consists of 5 elongated lanceolate scales, arranged in 3 parts (2 + 1 + 2), each of the parts are separated by 2 or 3 smaller scales, the 3rd nuchal crest the largest; dorsal crest on body weakly and composing of one row of keeled scales in normal size, extending to the base of the tail, located between 2 parallels, keeled, and enlarged scale rows; dorsal crest and 2 enlarged, discontinuous scale rows, separated from one another by small scales; the scales of the dorsal crest separated from the nuchal crest by a gap of 6 small dorsals; the antehumeral fold present; scales around midbody 57; dorsal scales are heterogenous in size and shape; not arranged in regular rows; the smaller scales feebly keeled and larger scales moderately keeled on flank of body; keels on scales of the flanks are obliquely downward; ventrals heavily keeled; and no sharp transition from scales on flanks.

Slender limbs, covered with irregular, keeled scales; the scales on outer surfaces of the limbs strongly keeled, and weakly keeled on the inner surface; hind limbs longer and slightly stronger than forelimbs; palmar and pedal subdigital lamellae bicarinate with spinose mucrons, except the base of fingers and toes; 23 lamellae beneath Finger IV, 24 lamellae beneath Toe IV; subdigital lamellae of Toe III modified, preaxial keels moderately protruded and pointed, and postaxial keels gradually weakened; tail laterally compressed, slightly swollen at base, covered with strongly keeled scales, and the vertebral scales on tail partial enlarged.

**Figure 6:**
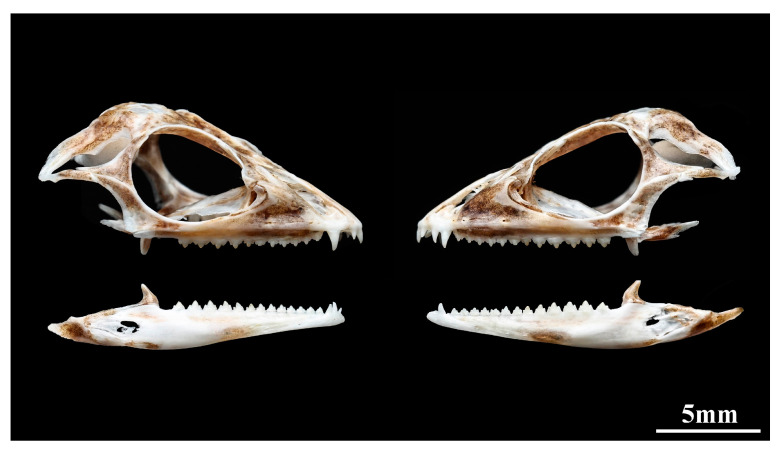
Lateral view of the skull of *Pseudocalotes jingpo* sp. nov. (Paratype, QHU2024003). Skeletal specimen was made by Junkang Hu. Scale bar = 5 mm.

Dentition: Premaxillary teeth 3, pleurodont, the middle one is the largest, and the smaller on both sides (Figure 6). Maxillary teeth 14/14, clearly divided into two distinct groups. The anterior part consists of two pleurodont teeth, approximately canine shaped, tip slightly curved backwards, the first one smaller while the second one is significantly enlarged (as well as the largest tooth). The posterior part consists of 12 acrodont tricuspid teeth, with the anterior 7 small and the posterior 5 enlarged. Among them, the 11th–13th is significantly enlarged, and the 10th and 14th are only slightly enlarged. Mandibular teeth 16, with a composition similar to that of the maxillary teeth. The anterior part consists of three thin and sharp pleurodont teeth, the first one small, while the second and thirs enlarged. The posterior part consists of 13 acrodont tricuspid teeth, with the anterior 8 small and the posterior 5 enlarged. Among them, the 13th–15th is significantly enlarged, and the 12th and 16th only slightly enlarged. The upper and lower teeth are alternating, i.e., maxillary teeth fit into a gap in the dentary and vice versa.

Coloration in life: In the light phase, the background coloration of the dorsal head, body, limbs, and tail is light taupe, scattered with dark brown irregular spots; three deep brown bands on on the dorsal surface of the head; multiple black brown short lines around the eyes form the radial stripe pattern; four diffused, irregular, brownish patches between limb insertions, not extending to ventral edge of flanks, the final patches incomplete and thin, over the pelvic region; nine bands on tail; small brown spots or irregular black brown rings scattered on the limbs, a light gray, irregular patch on each elbow and knee. Ventral surface of head ivory, gular region whitish and bearing brownish, oblique lines; gular spot absent; Venter ivory with light brown irregular oblique lines on both sides, lines crossing each other, extend inward, and are separated in the middle of the venter. Inner lips wathet, tongue aurantiacus, remaining oral cavity mostly bluish black.

In the dark phase, the overall color pattern is similar to the light phase. The difference is that the background coloration of the dorsal head, body, limbs, and tail is tan, scattered with blackish brown and fawn irregular spots, the irregular spots of lighter and darker coloration gives the anterior part of flank almost a reticulated appearance; as the color deepens, the reticulated pattern gradually blurs out; an oblique, white band below the eyes, extends to the venter surface of head; the dark patches on the dorsal body extend slightly to the flank, blend with the background color and almost invisible; a white, irregular patch on each elbow and knee. The brownish background color extends towards the venter, venter head and body light brown, gradually becoming lighter towards the middle; the brownish lines on the ventral surface of head and body further deepen.

### 3.5. Variation

Paratypes closely approximates the holotype in overall body coloration and pattern, except the postrostrals 5–6 (*n* = 4), cicrcumorbitals 8–12 (*n* = 4), canthals 5–7 (*n* = 4), supralabials 5–7 (*n* = 4), infralabials 6–8 (*n* = 4), enlarged scales between eye and ear 1–3 (*n* = 4), enlarged posttemporals 1–2 (*n* = 4), nuchal crest 3–5 (*n* = 4), postmentals 3–4 (*n* = 4), gulars 42–53 (*n* = 4), scales around midbody 53–62 (*n* = 4), Finger IV subdigital lamellae 20–26 (*n* = 4), Toe IV subdigital lamellae 24–29 (*n* = 4). Measurements and scalation features of the type series (*n* = 4) are presented in Table 4.

### 3.6. Comparisons

*Pseudocalotes jingpo* sp. nov. can be differentiated from all other species of *Pseudocalotes* by having the combination of following characters: interoculabials; canthals; cicrcumorbitals; visible parietal eye; superciliaries; supralabials; infralabials; the presence of a transverse gular fold and antehumeral fold; the number of enlarged scales between eye and ear, number of enlarged posttemporals; the number of nuchal crest and dorsal crest rows; dorsal body scales heterogenous in size and shape; light taupe dorsal background coloration with four irregular brown patches along the middle of dorsal and numerous other characteristics (Table 4).

**Figure 7:**
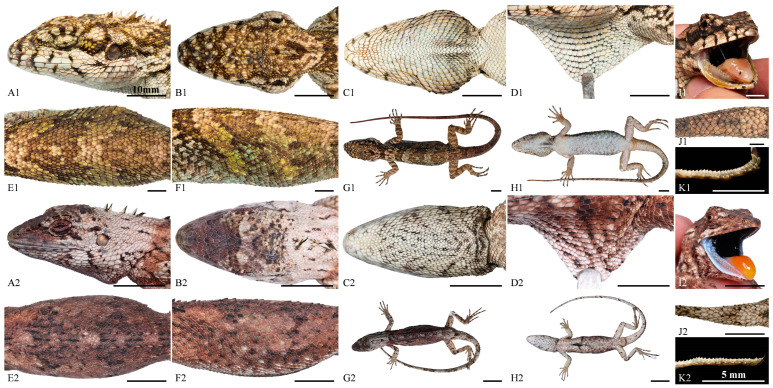
Comparisons of lateral (**A**), dorsal (**B**), ventral (**C**) views of the head, close-up view of the dewlap (**D**), dorsal I, and lateral (**F**) view of body, general view of dorsal (**G**) and ventral (**H**), close-up view of the oral cavity (**I**), the base of dorsal tail (**J**) and the lateral view of preaxial scales on the third toe (**K**) among *P. kakhienensis* (LFR2024001; topotype; adult female) (**A1**–**K1**) and *P. jingpo* sp. nov. (QHU2024002; holotype; adult female) (**A2**–**K2**). Scale bars: (**A1**–**K1,A2**–**J2**) = 10 mm; (**K2**) = 5 mm.

*Pseudocalotes jingpo* sp. nov. is most similar to its sister species *P. kakhienensis*. However, the new species can be distinguished from *P. kakhienensis* by having the 1st supralabial in contact with nasal (vs. 2 supralabials in contact with nasal); nuchal crest single, consists of 3–5 erected spines (vs. 7–13); transverse gular fold present (vs. absent); interoculabials 3–4 (vs. 2); the smaller dorsal scales feebly keeled and larger scales moderately keeled on flank of body; keels on scales of the flanks are obliquely downward (vs. the smaller dorsal scales moderately keeled and larger scales feebly keeled on flank of body, keels on those of the upper flanks are oriented obliquely upward, horizontal on the mid flanks and obliquely downward on the lower flanks); dorsal crest row discontinuous, located between two parallel and enlarged scale rows (vs. dorsal crest rows continuous, scale rows adjacent dorsal crest not enlarged); SVL up to 69.3 mm in female (vs. SVL up to 117 mm in female); inner lips wathet; tongue aurantiacus; the remaining oral cavity mostly bluish black (vs. inner lips aurantiacus, tongue flesh, remaining oral cavity mostly black); and the markedly different dorsal color patterns. For more detailed information and visual comparisons, please refer to Table 5 and Figure 7.

*Pseudocalotes jingpo* sp. nov. differs from *P*. *microlepis* by having smooth gulars (vs. keeled); antehumeral fold present (vs. absent); enlarged supratympanics 1 (vs. absent); nuchal crest single, consists of 3–5 erected spines (vs. nuchal crest 8–10, with no gaps); scales around midbody 53–62, dorsal crest row discontinuous, located between two parallel and enlarged scale rows (vs. dorsal crest rows continuous, scale rows adjacent dorsal crest not enlarged); dorsal scales are heterogenous in size and shape (vs. scales around midbody 62–74, dorsal scales small and weakly keeled); ventrals smaller than dorsals (vs. ventrals almost equal to dorsals); Finger IV lamellae 20–26, and Toe IV lamellae 24–29 (vs. Finger IV lamellae 18–19, and Toe IV lamellae 21–25); and tail comparatively short, TAL/SVL ratio 1.32–1.48 in female (vs. tail long, TAL/SVL ratio 2.01–2.29 in female).

*Pseudocalotes jingpo* sp. nov. differs from *P. brevipes* by having smooth gulars (vs. keeled); a single nuchal crest consists of 3–5 erected spines (vs. 7–13); scales around midbody 53–62 (vs. 66–76); Finger IV lamellae 20–26 (vs. Finger IV lamellae 16–21); dorsal crest row discontinuous, located between two parallel and enlarged scale rows (vs. dorsal crest rows continuous, scale rows adjacent dorsal crest not enlarged); dorsal scales in irregular rows (vs. dorsal scales in regular rows); and the vertebral scales on tail not enlarged (vs. the vertebral scales on tail strongly keeled and enlarged).

*Pseudocalotes jingpo* sp. nov. differs from *P. kingdonwardi* by having postrostrals 5–6 (vs. 3–4); the presence of a transverse gular fold (vs. absent); nuchal crest single, consists of 3–5 erected spines (vs. 8–11); scales around midbody 53–62 (vs. 42–54); gulars 42–53 (vs. 36); dorsal crest row discontinuous, located between two parallel and enlarged scale rows (vs. dorsal crest rows continuous, scale rows adjacent dorsal crest not enlarged); and SVL up to 69.3 mm, TAL/SVL ratio 1.32–1.48 in female (vs. SVL up to 103.1mm, TAL/SVL ratio 2.02 in female).

With two other congeners that have irregular dorsal scale rows, *Pseudocalotes jingpo* sp. nov. differs from *P. flavigula* (Smith, 1924) by having scales around midbody 53–62 (vs. 38–44); interparietal present (vs. absent); dorsal crest row discontinuous, located between two parallel and enlarged scale rows (vs. dorsal crest rows continuous, scale rows adjacent dorsal crest not enlarged); and from *P. poilani* (Bourret, 1939) by having supralabials 5–7, infralabials 6–8 (vs. supralabials 8–9, infralabials 9–10), nuchal crest single, consists of 3–5 erected spines (vs. 7–9), and Finger IV lamellae 20–26 (vs. 18).

*Pseudocalotes jingpo* sp. nov. can be easily distinguished from *P. tympanistriga* (Gray, 1831), *P. andamanensis* (Boulenger, 1891), *P. floweri* (Boulenger, 1912), *P. saravacensis* Inger and Stuebing, 1994, *P. larutensis* Hallermann and Mcguire, 2001, *P. dringi* Hallermann and Böhme, 2000, *P. khaonanensis* Chan-ard, Cota, Makchai and Laoteow, 2008, *P. ziegleri* Hallermann, Truong, Orlov and Ananjeva, 2010, *P. cybelidermus* Harvey, Hamidy, Kurniawan, Shaney and Smith, 2014, *P. guttalineatus* Harvey, Hamidy, Kurniawan, Shaney and Smith, 2014, *P. rhammanotus* Harvey, Hamidy, Kurniawan, Shaney and Smith, 2014, *P. drogon* Grismer, Quah, Wood, Anuar, Muin, Davis, Murdoch, Grismer, Cota and Cobos, 2016, *P. rhaegal* Grismer, Quah, Wood, Anuar, Muin, Davis, Murdoch, Grismer, Cota and Cobos, 2016, *P. viserion* Grismer, Quah, Wood, Anuar, Muin, Davis, Murdoch, Grismer, Cota and Cobos, 2016, and *P. baliomus* Harvey, Shaney, Hamidy, Kurniawan and Smith, 2017 by having dorsals forming irregular rows and being heterogenous in size and shape (vs. dorsals forming regular rows). Moreover, the new species can be distinguished from *P. tympanistriga*, *P. rhammanotus*, *P. floweri*, *P. dringi*, *P. khaonanensis*, *P. saravacensis*, *P. drogon*, and *P. viserion* by having scales around midbody 53–62 (vs. 46–40 in *P. tympanistriga*, 51 in *P. rhammanotus*, 44 in *P. floweri*, 48–52 in *P. dringi*, 72–75 in *P. khaonanensis*, 68 in *P. saravacensis*, 51 in *P. drogon*, and 35–38 in *P. viserion*), from *P. andamanensis*, *P. larutensis*, *P. cybelidermus*, *P. guttalineatus* and *P. rhaegal* by having transverse gular fold present (vs. the transverse gular fold absent), from *P. ziegleri* and *P. baliomus* by having antehumeral fold present (vs. antehumeral fold absent).

### 3.7. Etymology

The specific epithet, *Pseudocalotes jingpo* sp. nov., is a Latinized noun in apposition, and is invariable, based on the distribution of this new species being similar to one of the minorities in China, the Jingpo Ethnic Group, which inhabit the mountain regions in southwest China, and the type locality Dehong Dai and Jingpo Autonomous Prefecture is also the concentrated area of the Jingpo Ethnic Group in China. We suggest Jǐng Pō Nǐ Shù Xī (景颇拟树蜥) as a Chinese common name and Jingpo False Garden Lizard as an English common name.

### 3.8. Distribution and Habitat

At present, this new species is only found at the type locality of Yingjiang County, Dehong Dai and Jingpo Autonomous Prefecture, Yunnan Province, China. We found the lizards on a clear day in the bushes by the roadside, with an air temperature of about 20 °C. The habitat environment was well-preserved tropical montane rainforest, at the elevation of approximately 1000 m.

## 4. Discussion

The subfamily Draconinae represent a remarkable radiation of reptiles distributed throughout Asia and Oceania, with more than 273 valid species recognized. However, despite the high diversity of this vast group, the intergeneric similarities and the morphological diversity of intrageneric species led to the unclear classification relationships among many genera. Morever, since many species in this group are still poorly understood, making it difficult to obtain samples, this results in the lack of phylogenetic research and difficult to proceed with the revisionary work.

The teeth of Draconinae are characterized by acrodont tricuspid dentition, and the dentition is also considered one of the important morphological characters for solving the intergeneric and interspecific relationships [38]. However, the dentition of the vast majority of species in the subfamily Draconinae is still unknown, and there is very little research on this aspect. In this study, we provided relevant data on the dentition of the new species. However, due to the lack of data, we are unable to determine the true characteristics of the dentition of different genera of Draconinae, and even unable to compare the differences among other species of the genus *Pseudocalotes*. Therefore, in the future, we need to strengthen international cooperation and conduct a large number of sample comparisons to help us further solve the classification relationships between different groups of the Draconinae.

The agamid lizards of genus *Pseudocalotes* has long been considered as an enigmatic assemblage of species. Although the genus has a broad distribution in southeast Asia, most of species restricted to montane refugia and have well concealment, so that it is hard to detect them in the wild, and many species were described based on a few or even a single type specimen (e.g., *P. rhammanotus* and *P. drogon*), and several species (e.g., *P. poilani* and *P. saravacensis*) do not have any sequence data accessioned [1,2,3,4]. Therefore, although some previous studies have reviewed and revised classification for the genus to some extent, but due to the lack of broader genetic and morphological sampling, we are still not clear enough about the classification status, and evolution history within this genus [1,3,4,5,7,20,21].

In this study, we combined the morphological and molecular phylogenetical analysis of the specimens in the genus *Pseudocalotes*. The ML tree that was generated from the dataset inferred the phylogenetic relationships: ((*Gonocephalus*, *Bronchocela*), *Pseudocalotes*). This result differs from the results obtained by Harvey et al., 2017 [6]. The genus *Pseudocalotes* was strongly suggested as a sister taxon to the genus *Gonocephalus* and *Bronchocela* (SH 97/UFB 96). The genus *Pseudocalotes* is a paraphyletic clade, the genus *Dendragama* inserted into it. Within genus *Pseudocalotes* is divided into two unrelated clades with low support: the clade 1 undoubtedly includes the vast majority of species in the genus *Pseudocalotes*; but the clade 2, which contains *P. Dioidema, P. guttalineatus*, *P. cybelidermus*, *P. baliomus*, *P*. *rhammanotus* and *P. tympanistriga*, is more closely related to the genus *Dendragama*. The phylogenetic relationships between the genus *Gonocephalus*, *Bronchocela*, *Pseudocalotes*), and *Dendragama* remained essentially unresolved. Meanwhile, our analyses clearly assigned the phylogenetic position of the newly collected specimens to the genus *Pseudocalotes*, four specimens of the new species from Yingjiang County, Yunnan Province were nested within the clade 1, formed a sister taxon with *P. kakhienensis* (SH 97/UFB 100).

Based on molecular evidence, *Pseudocalotes jingpo* sp. nov. is closely related to *P. kakhienensis*, but still possess a considerable level of genetic divergence from 14.5% to 16.3% in ND2 and 15.5% to 15.8% in ND4 to each other. Interestingly, the distribution pattern among two species significantly overlaps, and the type locality is adjacent to each other (Figure 1), but they can be distinguished easily by significant morphological features, so that we can be certain that it is a newly discovered lineage.

The description of *Pseudocalotes jingpo* sp. nov. brings the total number of *Pseudocalotes* species to 22. Yingjiang County, which is the type locality of the new species, is one of the areas with the most biodiverse in China. In recent years, with the progress of field surveys, many new species have been discovered here. Therefore, additional surveys may help the understanding of the biodiversity along the southwest China. Currently, the new species known only from the locality investigated, but the Yingjiang County is close to the borders of Myanmar, and this species may also occur in the adjacent area.

In China, the genus *Pseudocalotes* is mainly distributed in south of China mainland and Hainan Island. However, due to the bright body coloration pattern is typically in male, species of genus *Pseudocalotes* were often captured and traded as pets. In order to protect these beautiful species, this genus should be considered to include in the local protected animal lists to prohibit the pet trading.

## 5. Conclusions

We described a new species of the genus *Pseudocalotes*, *Pseudocalotes jingpo* sp. nov., based on four female specimen collected from Yingjiang County, Dehong Dai and Jingpo Autonomous Prefecture, Yunnan Province, China. Since Yingjiang County is close to the borders of Myanmar, this species may also occur in the adjacent area. However, due to their cryptic lifestyle, the discovery of the new species is largely accidental, which makes it difficult for us to make accurate judgments on the distribution and population status of this species. Further research is needed to elucidate the true distribution range and ecological niche of the new species.

## Figures and Tables

**Table 1:** GenBank accession numbers, localities, and voucher information for all specimens used in this study.

ID	Species Name	Locality	Voucher	ND2	ND4	Reference
1	*Pseudocalotes jingpo* sp. nov.	Yingjiang, Yunnan, China	QHU2023033	PP228248	PP356075	This study
2	*P. jingpo* sp. nov.	Yingjiang, Yunnan, China	QHU2023040	PP228249	PP356076	This study
3	*P. jingpo* sp. nov.	Yingjiang, Yunnan, China	QHU2023041	PP228250	PP356077	This study
4	*P. jingpo* sp. nov.	Yingjiang, Yunnan, China	QHU2023042	PP228251	PP356078	This study
5	*P. baliomus*	Sumatra Barat, Indonesia	ENS 14429	MT295830	MT295830	Shaney et al., 2020 [21]
6	*P. brevipes*	unknown	MVZ 224106	AF128502	—	Macey et al., 2000 [15]
10	*P. cybelidermus*	Sumatra Selatan, Indonesia	UTA R 60551	—	KT180139	Shaney et al., 2017 [18]
11	*P. cybelidermus*	Sumatra Selatan, Indonesia	UTA R 60549	—	KT180140	Shaney et al., 2017 [18]
12	*P. drogon*	Fraser’s Hill, Malaysia	LSUHC 12223	KX258211	—	Grismer et al., 2016 [5]
13	*P. flavigula*	Cameron Highlands, Malaysia	LSUHC 12420	KX258212	—	Grismer et al., 2016 [5]
14	*P. flavigula*	Cameron Highlands, Malaysia	LSUHC 12580	KX258213	—	Grismer et al., 2016 [5]
15	*P. floweri*	Bokor Plateau, Cambodia	LSUHC 8572	KX258215	—	Grismer et al., 2016 [5]
16	*P. guttalineatus*	South Sumatra, Indonesia	ENS 14712	MT295833	MT295833	Shaney et al., 2020 [21]
17	*P. kakhienensis*	unknown	CAS242688	—	KY884006	Harvey et al., 2017 [6]
18	*P. kakhienensis*	Gongshan, Yunnan, China	KIZ 015975	MK001435	—	Wang et al., 2019 [7]
19	*P. kakhienensis*	Yingjiang, Yunnan, China	LFR2024001	PP228252	PP356079	This study
20	*P. kakhienensis*	Yingjiang, Yunnan, China	LFR2024002	PP228253	PP356080	This study
21	*P. kingdonwardi bapoensis*	Dulongjiang, Yunnan, China	CAS 241966	MK001436	—	Wang et al., 2019 [7]
22	*P. kingdonwardi bapoensis*	Dulongjiang, Yunnan, China	CAS 242628	MK001437	—	Wang et al., 2019 [7]
23	*P. larutensis*	Bukit Larut, Malaysia	LSUHC 9041	—	KY884012	Harvey et al., 2017 [6]
24	*P. larutensis*	Bukit Larut, Malaysia	LSUHC 9052	KX258219	KY884013	Grismer et al., 2016 [5], Harvey et al., 2017 [6]
25	*P. larutensis*	Bukit Larut, Malaysia	LSUHC 11289	KX258217	—	Grismer et al., 2016 [5]
26	*P. microlepis*	Hainan, China	XLHZ601	KX898132	KX898132	Yu et al., 2018 [20]
27	*P. rhaegal*	Cameron Highlands, Malaysia	LSUHC 12000	KX258220	—	Grismer et al., 2016 [5]
28	*P. rhaegal*	Cameron Highlands, Malaysia	LSUHC 12179	KX258221	—	Grismer et al., 2016 [5]
29	*P. rhammanotus*	Lampung, Sumatra, Indonesia	MZB 10804	—	KT180147	Shaney et al., 2017 [18]
30	*P. tympanistriga*	West Java, Indonesia	unknown	MT295858	MT295858	Shaney et al., 2020 [21]
31	*P. tympanistriga*	West Java, Indonesia	ENS16172	MT295859	MT295859	Shaney et al., 2020 [21]
32	*P. viserion*	Genting Highlands, Malaysia	LSUHC 12114	KX258222	—	Grismer et al., 2016 [5]
33	*P. viserion*	Genting Highlands, Malaysia	LSUHC 12227	KX258223	—	Grismer et al., 2016 [5]
34	*Bronchocela cristatella*	Selangor, Malaysia	TNHC 57874	AF128495	—	Macey et al., 2000 [13]
35	*B. cristatella*	Lampung, Indonesia	UTA R 62895	—	KT180148	Shaney et al., 2017 [18]
36	*B. jubata*	Lampung, Indonesia	UTA R 62899	—	KT180146	Shaney et al., 2017 [18]
37	*Dendragama boulengeri*	West Sumatra, Indonesia	ENS 19642	—	MT316077	Shaney et al., 2020 [21]
38	*D. dioidema*	Aceh, Indonesia	ENS 19481	—	MT316076	Shaney et al., 2020 [21]
39	*Gonocephalus chamaeleontinus*	Tioman, Malaysia	LSUHC 3789	KX772856	—	Welton et al., 2017 [19]
40	*G. grandis*	Tioman, Malaysia	LSUHC 3836	KX772857	—	Welton et al., 2017 [19]
	**Out group**					
41	*Pogona vitticeps*	unknown	unknown	AB166795	NC006922	Amer and Kumazawa, 2005 [16]
42	*Calotes versicolor*	unknown	NUM: Az382	AB183287	AB183287	Amer and Kumazawa, 2007 [17]

**Table 2:** Uncorrected *p*-distances (%) among the *Pseudocalotes* species based on the partial mitochondria ND2 gene.

ID	Species	1–4	5	6	7	8–9	10	11	12–15	16–17	18–19	20	21–22	23–24	25–26
1–4	*P. jingpo* sp. nov.	0.3–2.1													
5	*P. baliomus*	32.8–33.4	–												
6	*P. brevipes*	21.1–22.3	30.1	–											
7	*P. drogon*	27.4–28.0	34.6	23.5	–										
8–9	*P. flavigula*	29.5–30.1	39.5	30.1	33.7	–									
10	*P. floweri*	26.5–27.1	32.8	25.3	26.5	37.1	–								
11	*P. guttalineatus*	31.0–31.9	22.0	30.4	29.5	39.5	31.6	–							
12–15	*P. kakhienensis*	14.5–16.3	31.0–33.1	20.5–21.4	23.5–25.1	29.8–31.6	23.2–25.0	28.3–29.5	0.3–5.9						
16–17	*P. kingdonwardi bapoensis*	22.3–23.5	30.7	21.4	24.4	31.9	25.0	29.2	19.0–20.8	–					
18–19	*P. larutensis*	23.5–24.4	33.1	19.3	11.1	33.1	23.5	31.0	21.4–23.8	20.2	–				
20	*P. microlepis*	20.5–21.7	30.7	6.3	22.9	30.7	25.3	31.3	19.3–21.4	20.8	18.7	–			
21–22	*P. rhaegal*	23.8–24.4	33.7	19.6	13.0	35.2	24.7	33.1	22.9–24.7	21.7	8.4	20.5	–		
23–24	*P. tympanistriga*	34.0–35.2	9.9–11.4	31.0	33.4–33.9	41.6	31.6–32.5	21.4–22.3	32.2–33.7	32.5	33.4–33.7	32.2	35.8–36.1	1.5	
25–26	*P. viserion*	26.2–26.8	35.5	25.9	29.2	21.7	31.6	33.4	23.2–25.0	29.5	30.1	25.6	31.9	35.8	–

**Table 3:** Uncorrected *p*-distances (%) among the *Pseudocalotes* species based on the partial mitochondria ND4 gene.

ID	Species	1–4	5	6	7–8	9	10–12	13–14	15	16	17–18
1–4	*P. jingpo* sp. nov.	0–0.85									
5	*P. baliomus*	23.9–24.5	–								
6	*P. brevipes*	17.5–18.1	24.0	–							
7–8	*P. cybelidermus*	23.2–23.5	15.5	24.0	–						
9	*P. guttalineatus*	23.9–24.2	17.2	25.2	9.4	–					
10–12	*P. kakhienensis*	15.5–15.8	22.8–23.7	19.3–19.8	22.7–23.7	23.2–24.1	1.8–5.6				
13–14	*P. larutensis*	19.4	25.0	18.6	22.0	21.1	17.2–18.2	–			
15	*P. microlepis*	16.2–16.7	23.7	6.5	23.5	23.9	17.7–18.6	17.5	–		
16	*P. rhammanotus*	23.7–24.0	9.0	23.2	15.5	15.8	22.8–24.0	25.2	24.2	–	
17–18	*P. tympanistriga*	24.2–25.0	9.5–9.9	25.2–25.4	16.2–16.5	16.0	23.3–24.9	25.4–25.6	25.6–25.7	7.8–8.2	0.34

**Table 4:** Main morphological characters of the type series of *Pseudocalotes jingpo* sp. nov.

	*Pseudocalotes jingpo* sp. nov.			
	QHU2024002 Holotype	QHU2024001 Paratype	QHU2024003 Paratype	QHU2024004 Paratype
Collection number	LFR2023040	LFR2023033	LFR2023041	LFR2023042
Sex	female	female	female	subadult female
SVL	57.5	69.3	56.1	35.1
TAL	85.3	96.1	74.9	46.3
TAL/SVL	1.48	1.38	1.34	1.32
HL	15.1	18.3	14.5	10.5
HW	8.9	10.4	8.6	6.3
HW/HL	0.59	0.57	0.59	0.6
Postrostrals	6	6	5	5
Cicrcumorbitals	11/12	10/11	9/10	8/10
Canthals	6/7	6/6	6/6	5/5
Superciliaries	6/5	6/6	5/5	6/5
Interparietal	1	1	1	1
Scales between rostral and nasal	1	1	1	1
Interoculabials	4	4	3	3
Supralabials contacting nasal	1	1	1	1
Supralabials	7/7	6/6	6/6	5/6
Infralabials	7/8	7/6	7/6	6/6
Postmentals	4	3	3	3
Chin-shields	5/4	5/5	4/5	4/4
Gulars	53	42	50	48
Transverse gular fold	present	present	present	present
Antehumeral fold	present	present	present	present
Enlarged scales between the eye and ear	2/2	2/2	1/2	3/2
Enlarged posttemporals	1	2/1	1/2	1
Enlarged supratympanic	1	1	1	1
Enlarged postrictals	0	0	0	0
Nuchal crest	5	3	5	5
Dorsal crest rows	discontinuous	discontinuous	discontinuous	discontinuous
Dorsal scales in size and shape	heterogenous	heterogenous	heterogenous	heterogenous
Flank scales keeled	yes	yes	yes	yes
Scales around midbody	57	62	53	53
Midventrals smaller than dorsals	yes	yes	yes	yes
Finger IV subdigital lamellae count	23	26	21	20
Toe IV subdigital lamellae count	24	29	27	28
Preaxial lamellae on toe III	modified	modified	modified	modified
Gular spot	absent	absent	absent	absent

**Table 5:** Morphological characters of *Pseudocalotes* obtained from specimens examined in this study and literature.

	*P. jingpo* sp. nov.	*P. andamanensis*	*P. baliomus*	*P. brevipes*	*P. cybelidermus*	*P. dringi*	*P. drogon*	*P. flavigula*
Interoculabials	3–4	/	3–4	/	2	/	/	2
Cicrcumorbitals	8–12	11	10	9–13	9–13	/	11	10–11
Canthals	5–7	4–5	6–7	5–7	5–6	5	5	4–5
Superciliaries	5–6	7–8	6–8	5–7	4–7	/	7–10	5–7
Parietal eye visible or invisible	visible	visible	invisible	visible	invisible	/	invisible	/
Postrostrals	5–6	4–6	5–6	5–7	4–6	5	7	4–6
Scales between rostral and nasal	yes	yes	yes	variable	variable	no	yes	no
Supralabials contacting nasal	1	yes	no	0–1	0–2	yes	yes	1
Supralabials	5–7	9–10	8–11	6–10	7–10	8	9	6–10
Infralabials	6–8	10–12	8–10	7–10	7–9	7–8	8	7–9
Postmentals	3–4	2	/	1–2	2–5	2	4	3–4
Gulars	42–53	/	41–42	/	/	/	47	40–46
Gulars smooth or keeled	smooth	keeled	keeled	keeled	keeled	smooth	smooth	smooth
Transverse gular fold	present	absent	present	present	absent	absent	present	present
Antehumeral fold	present	present	absent	present	absent	absent	present	present
Enlarged scales between the eye and ear	2–3	3–5	4–5	1–4	1–4	2	2	2–3
Enlarged posttemporals	1–2	present	present	1–2	1–2	present	1	2
Enlarged supratympanic	1	absent	/	variable	present	absent	absent	absent
Enlarged postrictals	absent	absent	absent	absent	present	absent	absent	absent
Nuchal crest	3–5	11–14	10–11	7–13	9–13	6	8	5–10
Dorsals in irregular or regular rows	irregular	regular	regular	irregular	regular	regular	regular	irregular
Dorsal crest rows	discontinuous	/	continuous	continuous	/	/	continuous	/
Flank scales keeled	yes	no	yes	yes	yes	yes	yes	yes
Scales around midbody	53–62	57–62	53–55	66–76	51–65	48–52	51	41–44
Midventrals smaller than dorsals	yes	yes	no	no	no	yes	yes	yes
Finger IV subdigital lamellae count	20–26	/	22–26	16–21	22–29	20	19	22–28
Toe IV subdigital lamellae count	24–29	27–30	28–30	20–27	24–30	26	23	26–30
Preaxial lamellae on toe III	modified	modified	subequal	serrate fringe Fringe	basally	not modified	enlarged, sharp	not modified
Dewlap pattern in adult male	/	yellow/white	no gular spot	purple or tawny	light green with cyan center	purple	lime-green w/yellow center	yellow
	***P. floweri***	***P. guttalineatus***	***P. kakhienensis***	***P. khaonanensis***	***P. kingdonwardi***	***P. larutensis***	***P. microlepis***	
Interoculabials	/	2	2	/	/	3	3	
Cicrcumorbitals	10–12	9–13	9–12	/	7–10	11–14	10	
Canthals	5–6	5–7	4–7	6	4–6	4–6	5–7	
Superciliaries	8	5–8	5–7	8	4–6	6–8	6–7	
Parietal eye visible or invisible	/	invisible	visible	/	visible	/	visible	
Postrostrals	4–6	5–7	5–7	/	3–4	5–7	6–7	
Scales between rostral and nasal	variable	no	variable	yes	variable	yes	no	
Supralabials contacting nasal	1–2	0–2	2	yes	1–2	yes	1	
Supralabials	8–10	9–10	7 –8	8	6–8	8–10	7–8	
Infralabials	9–11	7–10	6–8	9	6–8	8–10	7–9	
Postmentals	1–2	2–4	1–3	/	2–3	3–4	1	
Gulars	42	/	42–48	/	36	55–69	/	
Gulars smooth or keeled	smooth	keeled	smooth	/	smooth	keeled	keeled	
Transverse gular fold	present	absent	absent	absent	absent	absent	present	
Antehumeral fold	present	absent	present	present	present	present	absent	
Enlarged scales between the eye and ear	2–4	2–3	2–3	1	1–3	2–3	1–4	
Enlarged posttemporals	1	1	1	1	2–3	1	1	
Enlarged supratympanic	absent	present	present	absent	present	absent	absent	
Enlarged postrictals	absent	present	variable	absent	absent	absent	absent	
Nuchal crest	9	10–12	7–13	9	8–11	5–9	8–10	
Dorsals in irregular or regular rows	regular	regular	irrigular	regular	irregular	irregular	regular	
Dorsal crest rows	/	/	continuous	/	continuous	/	continuous	
Flank scales keeled	yes	yes	yes	yes	yes	yes	yes	
Scales around midbody	44	45–55	59–66	72–75	42–54	52–55	62–74	
Midventrals smaller than dorsals	yes	yes	yes	no	yes	yes	no	
Finger IV subdigital lamellae count	21–22	22–28	20–22	21	20–24	17–20	18–19	
Toe IV subdigital lamellae count	24–25	26–32	23–27	27	23–27	22–25	21–25	
Preaxial lamellae on toe III	serrate fringe	basally	modified	not modified	basally	not modified	serrate fringe	
Dewlap pattern in adult male	purple	grayish blue lines	oblique lines	purple	no color potch	yellow/purple	red or brown	
	***P. poilani***	***P. rhaegal***	***P. rhammanotus***	***P. saravacensis***	***P. tympanistriga***	***P. viserion***	***P. ziegleri***	
Interoculabials	/	2	2	/	2	2	/	
Cicrcumorbitals	10–12	9–10	9–10	/	9–13	10	/	
Canthals	6–7	5	6	4	5–6	4–5	5–7	
Superciliaries	5–9	7–10	5–7	/	6–8	5–7	/	
Parietal eye visible or invisible	/	visible	invisible	/	/	visible	/	
Postrostrals	7–8	6–8	5	/	4–7	3	7	
Scales between rostral and nasal	no	yes	no	yes	no	no	no	
Supralabials contacting nasal	1	yes	2	yes	1–2	yes	yes	
Supralabials	8–9	8–9	9	7	9–11	6–7	8–11	
Infralabials	9–10	7–8	8	7	9–11	6–7	7–11	
Postmentals	1–2	4	4–5	2	2–5	2–3	2–3	
Gulars	/	40–45	/	/	/	47–48	/	
Gulars smooth or keeled	smooth	smooth	keeled	keeled	keeled	smooth	smooth	
Transverse gular fold	present	absent	absent	absent	absent	present	absent	
Antehumeral fold	present	present	present	absent	present	present	/	
Enlarged scales between eye and ear	1–3	3–4	2	3	3–4	2	2	
Enlarged posttemporals	3–4	1	1	2	1	1	absent	
Enlarged supratympanic	present	absent	present	present	present	absent	/	
Enlarged postrictals	absent	absent	absent	absent	absent	absent	absent	
Nuchal crest	7–9	6–8	8	7	4–11	7–9	6–7	
Dorsals in irregular or regular rows	irregular	regular	regular	regular	regular	regular	regular	
Dorsal crest rows	/	continuous	/	/	/	continuous	continuous	
Flank scales keeled	yes	yes	yes	yes	no	yes	no	
Scales around midbody	54–62	52–58	51	68	40–46	35–38	57–64	
Midventrals smaller than dorsals	yes	yes	yes	/	yes	yes	yes	
Finger IV subdigital lamellae count	18	19–21	21	16	22–26	22–23	/	
Toe IV subdigital lamellae count	23	22–26	24	20	26–32	26–27	24	
Preaxial lamellae on toe III	serrate fringe	enlarged, rounded	not modified	/	not modified	not modified	modified	
Dewlap pattern in adult male	/	cyan w/purple center	brown with white center	black speckles	same color as venter	yellow	blue	

## Data Availability

The data presented in this study are available on request from the corresponding author. ZooBank Code: urn:lsid:zoobank.org:act:FBF1A402-DF7F-44BC-878C-50C5548AD32B; urn:lsid:zoobank.org:pub:EECB346B-B738-42CA-A06C-386CC9253B1F.
